# Emergence of *SCCmec* variants causing false-negative MRSA results by Xpert SA Nasal Complete: a need for culture back-up?

**DOI:** 10.1128/asmcr.00069-26

**Published:** 2026-07-23

**Authors:** Amy L. Leber, Sophonie J. Oyeniran, Jonatan Lehtinen, Huanyu Wang

**Affiliations:** 1 Department of Pathology and Laboratory Medicine, Nationwide Children’s Hospital, Columbus, Ohio, USA; 2 Department of Pathology, The Ohio State University 2647, Columbus, Ohio, USA; 3 Solu Healthcare Oy, Helsinki, Finland; Pattern Bioscience, Austin, Texas, USA

**Keywords:** MRSA screening, *Staphylococcus aureus*, molecular typing

## Abstract

**Background:**

*Staphylococcus aureus* (SA) is a major human pathogen and an important cause of healthcare-associated infections. Hospital-acquired methicillin-resistant *S. aureus* (MRSA) is associated with increased morbidity and mortality. Screening for nasal carriage of SA followed by decolonization has been shown to reduce healthcare-associated MRSA transmission and infection. Nucleic acid amplification assays (NAATs) are widely used for MRSA screening and are associated with shorter turnaround times, fewer isolation days, reduced MRSA-related infections, and improved clinical outcomes and cost savings.

**Case Summary:**

Two patients underwent preoperative nasal screening using Xpert SA Nasal Complete, and corresponding culture results were discordant with the molecular results. In both cases, the Xpert SA assay reported “SA detected and MRSA not detected,” whereas culture and antimicrobial susceptibility testing (AST) demonstrated the presence of MRSA. Additional testing supported the culture-based identification of MRSA. Whole-genome sequencing and molecular typing revealed that both MRSA isolates harbored *SCCmec* variants that were not detected by the Xpert assay, leading to false-negative (FN) MRSA results.

**Conclusion:**

While NAATs remain highly sensitive and reliable tools for MRSA screening and the overall risk of FN MRSA detection may be low, our cases highlight the importance of ongoing surveillance of local MRSA epidemiology and understanding the genetic inclusivity of the molecular assays used for detection. In high-risk or targeted patients, consideration of reflex culture may be warranted.

## INTRODUCTION

Nasal colonization with methicillin-resistant *Staphylococcus aureus* (MRSA) is a recognized risk factor for subsequent infection and can spread within healthcare settings. It is associated with increased morbidity and mortality. Identification of nasal *Staphylococcus aureus* (SA) colonization followed by decolonization has been shown to decrease MRSA transmission and infection. The Cepheid Xpert SA Nasal Complete assay (Xpert SA, Cepheid, Sunnyvale, CA, USA) is a fully automated real-time PCR system that detects three SA targets: Staphylococcal protein A gene (*spa*) for SA identification, Staphylococcal cassette chromosome mec (*SCCmec*), and methicillin resistance (*mecA*), both of which must be detected for reporting methicillin resistance. Results are interpreted as SA and MRSA detected or not detected. The laboratory at Nationwide Children’s Hospital began offering this test in 2016 as part of selective preoperative evaluations. At our institution, testing involves collection of two nasal swabs: one is tested on Xpert SA and reported within 24 h, and the other is submitted for culture and phenotypic antimicrobial susceptibility testing (AST) if SA is recovered (1). Here, we report two cases in which Xpert SA detected SA only and culture recovered MRSA.

## CASE PRESENTATION

Patient A is a 4-year-old male with a complex cardiovascular disease history, who required prolonged hospitalization with multiple admissions to the intensive care unit (ICU). As part of preoperative screening, a nasal swab was tested, which resulted as “SA detected and MRSA not detected” by Xpert SA, with *spa* and *mecA* targets detected and *SCCmec* not detected. The companion culture, however, recovered both methicillin-susceptible SA (MSSA) and MRSA. Antimicrobial susceptibility testing on the isolates using Vitek 2 Gram-Positive Susceptibility Card (AST-GP75) demonstrated a negative screen for cefoxitin and an oxacillin minimum inhibitory concentration (MIC) of 0.5 µg/mL for MSSA and a positive screen for cefoxitin and oxacillin MIC ≥4 µg/mL for MRSA.

Patient B is a 1-month-old full-term male with a complete atrioventricular canal defect that required surgical intervention. As part of preoperative screening, his nasal swab resulted as “SA detected and MRSA not detected” by Xpert SA, with *spa* and *mecA* targets detected and *SCCmec* not detected; the companion culture grew only MRSA with a positive screen for cefoxitin and oxacillin MIC of ≥4 µg/mL.

The Xpert SA results on the nasal swabs and the AST results on the SA isolates for both patients are present in Table 1. Due to the discrepancy between genotypic and phenotypic findings, all cultured isolates underwent additional testing, including detection of penicillin-binding protein 2a (Alere PBP2a, Abbott, Chicago, IL, USA), and offline testing with a molecular blood culture identification system (BCID2, bioMérieux BioFire BCID 2, Salt Lake City, UT, USA); both confirmed the presence of MSSA in patient A and MRSA in patients A and B. The SA isolates were also tested off-label using Xpert SA, which failed to detect methicillin resistance in the two MRSA isolates (Table 1). All isolates underwent whole-genome sequencing. Sequencing reads were screened for contamination with Kraken2 v2.17.1 (PlusPF-16 database, 15 Oct 2025) and CheckM2 v1.0.1. The isolate from patient B was found to contain *Staphylococcus haemolyticus* reads. *S. aureus* reads were extracted with Kraken Tools v1.2.1 before assembly (2–4), and residual contaminant contigs were removed (5, 6). Multilocus strain typing (MLST) was performed using mlst v2.32.2 (Seemann, https://github.com/tseemann/mlst) with schemes from PubMLST (7), and *SCCmec* typing was performed using sccmec v1.2.0 (Petit, https://github.com/rpetit3/sccmec) with camlhmp and BLAST+ (8) through the Solu Platform (9). AMRFinderPlus v4.0.23 (database 2025-07-16.1) was used to identify all detectable resistance genes/mutations from the genome sequence (10). The MRSA isolate from patient A carried features of both *SCCmec* subtypes IId and IVg in an ST3606 background, whereas the isolate from patient B carried *SCCmec* type XIII in an ST152 background. The exact *SCCmec* structure of the isolate from patient’s A could not be fully resolved because the repeat-rich region was not spanned by short-read sequencing. These results are presented in Table 1. Additionally, the resistomes of all three isolates are presented in Table 2. Notably, isolates 1 and 2 from patient A exhibited identical resistant determinants, except that isolate 1 additionally carried the *mecA* gene.

**TABLE 1:** Xpert SA Nasal Complete, AST, and additional results for samples with discordant methicillin resistance^*a*^

Patient	Primary sample				SA isolate										
	Xpert SA Nasal Complete				AST (Vitek2 GP-75)			Xpert SA Nasal Complete				PBP2a	BCID2	MLST typing	*SCCmec* typing
	Target (Ct value)			Interpretation	Isolate	Cefoxitin screen	Oxacillin MIC (µg/mL)	Target (Ct value)			Interpretation				
	*spa*	*mecA*	*SCCmec*					*spa*	*mecA*	*SCCmec*					
Patient A	24.7	24.4	ND	SA detected MRSA not detected	1	Pos	≥4	15.1	15.1	ND	SA detected MRSA not detected	Positive	MRSA	ST-3606	Untypeable
					2	Neg	0.5	12.6	ND	ND	SA detected MRSA not detected	Negative	MSSA	ST-3606	N/A
Patient B	23.2	22.1	ND	SA detected MRSA not detected	1	Pos	≥4	13.7	14.2	ND	SA detected MRSA not detected	Positive	MRSA	ST-152	XIII

^*a*^ ND, not detected. AST, antimicrobial susceptibility testing; PBP2a, altered penicillin-binding protein; BCID2, molecular blood culture identification system; MLST, multilocus strain typing; N/A, not applicable.

**TABLE 2:** Antimicrobial resistance genes identified by whole-genome sequencing

Antimicrobial agents	Genes	Patient A, isolate 1	Patient A, isolate 2	Patient B, isolate 1
Amikacin, gentamicin, kanamycin, tobramycin	aac(6′)-Ie/aph(2′′)-Ia	ND^*a*^	ND	+^*b*^
Beta-lactam	blaI, blaZ	+	+	+
Chloramphenicol	catA	ND	ND	+
Clindamycin, erythromycin, streptogramin B, tylosin	Erm(C)	ND	ND	+
Erythromycin, spiramycin, telithromycin	Mph(C)	+	+	ND
Erythromycin, streptogramin B	Msr(A)	+	+	+
Fosfomycin	foxB, murA-G257D	+	+	ND
Kanamycin, tobramycin	aadD1	+	+	+
Lincosamide	lnu(A) vga(A)-LC	ND	ND	+
**Methicillin**	**mecA**	**+**	**ND**	**+**
Mupirocin	mupA	+	+	ND
Quinolone	gyrA_S84L, gyrA_S85P, parC_S80F, parE_D432V	+	+	ND
Sulfonamide	folP_E208K, folP_F17L	+	+	ND
Tetracycline	tet(38)	+	+	+
Tetracycline	tet(K)	ND	ND	+
Tigecycline	rpsJ_Y58D	+	+	NA
Trimethoprim	dfrG	+	+	+
Trimethoprim	dfrS1	ND	ND	+

^*a*^ ND, not detected.

^*b*^ +, detected.

## DISCUSSION

MRSA remains a major health threat among at-risk patient populations. Enhancing screening strategies to identify MRSA carriers at the time of hospital admission and timely implementation of appropriate precautions are crucial to limiting post-surgical infections and nosocomial transmission (11, 12). Rapid diagnostic tools are needed for early detection and containment of their spread. Studies have found that compared to culture-based screening, rapid NAAT-based screening for MRSA has been associated with shorter turnaround time, fewer isolation days, reduced MRSA-related infection, and improved clinical outcomes and cost savings (13, 14). Clinical trials have also demonstrated that NAAT-based MRSA screening resulted in early discontinuation of MRSA contact precautions and improved screening completion due to the ease of use (15).

However, a growing body of studies have suggested suboptimal performance of NAATs when used alone for nosocomial MRSA screening (16–19). One major cause of false-negative (FN) methicillin resistance can be attributed to the genetic variation in assay target regions, particularly within the *SCCmec*-orfX junction, a primary target in the NAATs that detects MRSA. *SCCmec* is a mobile genetic element containing *mecA* and related regulatory genes. It is highly diverse; various *SCCmec* elements have been reported since its initial characterization (20). Emerging or uncommon *SCCmec* variants may not be recognized by assay primers, leading to missed MRSA detection. There are several published reports of FN MRSA detection associated with Cepheid Xpert assays (Table 3). In one study by Laurent et al. that evaluates the Xpert MRSA assay, the sensitivity decreased to 69% due to failure to detect a specific *SCCmec* type IV variant (19). Sabat et al. described two FN results using Xpert MRSA NxG assay in the Netherlands; both involved isolates with novel *SCCmec* organization that precluded primer binding (21). Similarly, Monecke et al. reported an FN result with Xpert MRSA/SA BC in Austria caused by an insertion adjacent to orfX that interfered with *SCCmec* detection (22). In another study from the United States evaluating the Xpert SA Nasal Complete assay across multiple healthcare settings, two samples were identified as MSSA by Xpert SA Nasal Complete assay but grew MRSA in culture; genetic characterization of these SA isolates was not performed (16). FN methicillin resistance may also occur with low organism burden, below the assay’s limit of detection, or rarely, variation in the SA-specific targets, leading to complete assay failure for detection of both SA and MRSA (23, 24).

**TABLE 3:** Published reports of false-negative MRSA detection associated with Cepheid Xpert assays^*a*^

Reference	Assay	Target(s)	Origin(s) of the study	False-negative MRSA cases	MRSA confirmation method(s)	Proposed molecular mechanism
Laurent et al. (19)	Xpert MRSA assay	*SCCmec*	Belgium	4 isolates	Culture, phenotypic AST, PFGE, *spa* typing	*SCCmec* type IV variant not recognized by the assay
Yarbrough et al. (25)	Xpert MRSA NxG	*SCCmec*, *mecA/mecC*	Multi-site clinical trial (United States, Singapore, and Germany)	18 specimens	Culture, phenotypic AST	Not investigated; most false-negatives were attributed to MRSA burden below the assay limit of detection, and authors acknowledged the possibility of an unrecognized *SCCmec* variant not recognized by the assay
Sabat et al. (21)	Xpert MRSA NxG	*SCCmec*, *mecA/mecC*	Netherlands	2 isolates	Culture, phenotypic AST, whole-genome sequencing	Novel *SCCmec* organization precluded primer binding
FDA MAUDE adverse event report^*b*^	Xpert MRSA NxG	*SCCmec*, *mecA/mecC*	United States	1 isolate	Culture, phenotypic AST, sequencing analysis	Insertion within the *SCCmec*-chromosome junction separating assay primer/probe targets
Monecke et al. (22)	Xpert MRSA/SA BC	*spa* for SA, *mecA/SCCmec* for MRSA	Australia	1 isolate	Culture, phenotypic AST, whole-genome sequencing	Large insertion into the *orfx/SCCmec* integration site
Mura et al. (23)	Xpert MRSA/SA BC	*spa* for SA, *mecA/SCCmec* for MRSA	France	1 isolate	Culture, phenotypic AST, whole-genome sequencing	Deletions within the *spa* gene led to false-negative SA and MRSA
Patel et al. (16)	Xpert SA Nasal Complete	*spa* for SA, *mecA/SCCmec* for MRSA	United States	2 specimens	Culture, phenotypic AST	Not investigated
This study	Xpert SA Nasal Complete	*spa* for SA, *mecA/SCCmec* for MRSA	United States	2 isolates	Culture, phenotypic AST, whole-genome sequencing	*SCCmec* variants not detected by the assay

^*a*^ AST, antimicrobial susceptibility test; SA, *Staphylococcus aureus*.

^*b*^ Available at https://www.accessdata.fda.gov/scripts/cdrh/cfdocs/cfmaude/detail.cfm?mdrfoi__id=21599431&pc=NQX.

In our institution, companion bacterial culture is set up routinely alongside the Xpert SA assay since the clinical adoption of this assay. We previously reported an overall agreement of 94% between Xpert SA and culture in a cohort of 1,264 pediatric samples (1). In that study, one sample was falsely reported as MSSA while the culture grew MRSA. This was attributable to low bacterial load, resulting in an *mecA* Ct above the manufacturer’s cutoff for positive. Ongoing performance monitoring had not identified FN methicillin resistance due to *SCCmec* variation until 2025, when two such cases emerged. To the best of our knowledge, FN methicillin results due to the *SCCmec* variants described in our cases have not been reported.

Notably, the MSSA and MRSA isolates from patient A differed by only two single-nucleotide polymorphisms and shared the same MLST and identical resistomes except *mecA*, suggesting identical strains. This finding supports that this MSSA isolate arose from *SCCmec* or *mecA* excision from the MRSA isolate, a phenomenon that has been reported previously (26). Conversely, an MSSA strain may also acquire *SCCmec* through horizontal transfer from MRSA or methicillin-resistant coagulase-negative *Staphylococcus* (CoNS) (27, 28). Together, these observations highlight the dynamic nature of *SCCmec* mobility, the potential for *in vivo* interconversion between MSSA and MRSA, and its implications for laboratory testing, patient management, and infection control.

For Xpert SA, detection of both *mecA* and *SCCmec* is required for an MRSA result. In nasal specimens that frequently contain methicillin-resistant coagulase-negative *Staphylococcus* (CoNS), detection of *mecA* alone is not sufficiently specific for identification of MRSA. A *spa*+/*mecA*+/*SCCmec*− profile commonly reflects the presence of *mecA* in another organism such as CoNS, rather than SA itself, and it is interpreted as “SA detected and MRSA not detected” by the Xpert SA. In our cohort, 304 (19.9%) out of 1,526 nasal swabs collected between May 2023 and November 2025 yielded MSSA by culture. A total of 253 (83.2%) had an *spa*+/*mecA*+/*SCCmec*− molecular profile, indicating that this pattern is common and reflects mixed staphylococcal colonization in nasal specimens. However, in rare cases, this profile may also indicate an MRSA with a variant *SCCmec* not detected by the assay, resulting in FN methicillin-resistance results. In the two patients reported here, the *mecA* target was detected but the *SCCmec* was not, leading to misclassification of MRSA as MSSA.

It is likely that most laboratories do not set up a companion culture for every test when running an MRSA screening assay due to cost considerations and acknowledging the overall reliable performance for accurately detecting methicillin resistance. In settings where a companion culture and AST are not routinely performed, a conservative approach could include setting up a reflex culture from all or a subset of critical patients, allowing detection and surveillance for emerging *SCCmec* variants. Another possible strategy is to evaluate the difference in Ct values (ΔCt) between the *spa* and *mecA* targets and to perform cultures for samples with an *spa*+/*mecA*+/*SCCmec*− profile and a ΔCt between *spa* and *mecA* less than 2 since an MRSA strain would be expected to have equivalent copies of *spa*, *mecA,* and SCC-orf (18, 29). Both cases presented here exhibited a ΔCt of <2 (Table 1). During the study period, among the 253 nasal swabs with this molecular profile but MSSA by culture, 193 (76.3%) samples had ΔCt (*spa* and *mecA*) greater than 2. Sixty samples (23.7%) had a Ct less than 2, representing 3.9% (60/1,536) of all samples screened during the study period. The targeted culture on the samples with ΔCt less than 2 may be a reasonable and more targeted strategy for culture surveillance for possible MRSA *SCCmec* variants missed by NAAT. A similar ΔCt-based interpretation algorithm has been adopted by an FDA-cleared test (MRSA/SA ELITe MGB Assay) to determine the presence of MRSA from nasal swabs and was found to demonstrate excellent concordance with phenotypic AST (18). While NAATs remain highly sensitive, reliable tools, and the overall risk of FN MRSA results may be low, ongoing surveillance of local MRSA epidemiology, understanding of the inclusivity of the MRSA molecular test used, and consideration of reflex culture in high-risk patients are warranted to mitigate the impact of emerging variants.

## Data Availability

The assembled sequences of all three isolates have been submitted to the NCBI Genome database under BioSample accession numbers SAMN61037317, SAMN61037318, and SAMN61037319.

## References

[1] Wang H, Salamon D, Jean S, Leber AL. 2021. Evaluation of the Cepheid Xpert SA nasal complete for direct detection of *Staphylococcus aureus* and methicillin-resistant *Staphylococcus aureus* in nasal swabs from pediatric patients. Diagn Microbiol Infect Dis 101:115417. doi:10.1016/j.diagmicrobio.2021.115417 34116341

[2] Wood DE, Lu J, Langmead B. 2019. Improved metagenomic analysis with Kraken 2. Genome Biol 20:257. doi:10.1186/s13059-019-1891-0 31779668 PMC6883579

[3] Chklovski A, Parks DH, Woodcroft BJ, Tyson GW. 2023. CheckM2: a rapid, scalable and accurate tool for assessing microbial genome quality using machine learning. Nat Methods 20:1203–1212. doi:10.1038/s41592-023-01940-w 37500759

[4] Lu J, Rincon N, Wood DE, Breitwieser FP, Pockrandt C, Langmead B, Salzberg SL, Steinegger M. 2022. Metagenome analysis using the Kraken software suite. Nat Protoc 17:2815–2839. doi:10.1038/s41596-022-00738-y 36171387 PMC9725748

[5] Bankevich A, Nurk S, Antipov D, Gurevich AA, Dvorkin M, Kulikov AS, Lesin VM, Nikolenko SI, Pham S, Prjibelski AD, et al.. 2012. SPAdes: a new genome assembly algorithm and its applications to single-cell sequencing. J Comput Biol 19:455–477. doi:10.1089/cmb.2012.0021 22506599 PMC3342519

[6] Li H. 2018. Minimap2: pairwise alignment for nucleotide sequences. Bioinformatics 34:3094–3100. doi:10.1093/bioinformatics/bty191 29750242 PMC6137996

[7] Jolley KA, Bray JE, Maiden MCJ. 2018. Open-access bacterial population genomics: BIGSdb software, the PubMLST.org website and their applications. Wellcome Open Res 3:124. doi:10.12688/wellcomeopenres.14826.1 30345391 PMC6192448

[8] Camacho C, Coulouris G, Avagyan V, Ma N, Papadopoulos J, Bealer K, Madden TL. 2009. BLAST+: architecture and applications. BMC Bioinformatics 10:421. doi:10.1186/1471-2105-10-421 20003500 PMC2803857

[9] Saratto T, Visuri K, Lehtinen J, Ortega-Sanz I, Steenwyk JL, Sihvonen S. 2025. Solu: a cloud platform for real-time genomic pathogen surveillance. BMC Bioinformatics 26:12. doi:10.1186/s12859-024-06005-z 39806295 PMC11731562

[10] Feldgarden M, Brover V, Gonzalez-Escalona N, Frye JG, Haendiges J, Haft DH, Hoffmann M, Pettengill JB, Prasad AB, Tillman GE, et al.. 2021. AMRFinderPlus and the Reference gene catalog facilitate examination of the genomic links among antimicrobial resistance, stress response, and virulence. Sci Rep 11:12728. doi:10.1038/s41598-021-91456-0 34135355 PMC8208984

[11] Awad SS, Palacio CH, Subramanian A, Byers PA, Abraham P, Lewis DA, Young EJ. 2009. Implementation of a methicillin-resistant *Staphylococcus aureus* (MRSA) prevention bundle results in decreased MRSA surgical site infections. Am J Surg 198:607–610. doi:10.1016/j.amjsurg.2009.07.010 19887186

[12] Schelenz S, Tucker D, Georgeu C, Daly S, Hill M, Roxburgh J, French GL. 2005. Significant reduction of endemic MRSA acquisition and infection in cardiothoracic patients by means of an enhanced targeted infection control programme. J Hosp Infect 60:104–110. doi:10.1016/j.jhin.2004.11.020 15866007

[13] Pinsent A, Winter K, Chase J, Martinaud C, Nicolau DP, Folse H. 2026. Impact of meticillin-resistant *Staphylococcus aureus* rapid molecular testing on hospital-related quality and cost of care. J Hosp Infect 167:1–8. doi:10.1016/j.jhin.2025.09.013 41075839

[14] Polisena J, Chen S, Cimon K, McGill S, Forward K, Gardam M. 2011. Clinical effectiveness of rapid tests for methicillin resistant *Staphylococcus aureus* (MRSA) in hospitalized patients: a systematic review. BMC Infect Dis 11:336. doi:10.1186/1471-2334-11-336 22151575 PMC3259066

[15] Shenoy ES, Kim J, Rosenberg ES, Cotter JA, Lee H, Walensky RP, Hooper DC. 2013. Discontinuation of contact precautions for methicillin-resistant *Staphylococcus aureus*: a randomized controlled trial comparing passive and active screening with culture and polymerase chain reaction. Clin Infect Dis 57:176–184. doi:10.1093/cid/cit206 23572482 PMC3689342

[16] Patel PA, Schora DM, Peterson KE, Grayes A, Boehm S, Peterson LR. 2014. Performance of the Cepheid Xpert SA nasal complete PCR assay compared to culture for detection of methicillin-sensitive and methicillin-resistant *Staphylococcus aureus* colonization. Diagn Microbiol Infect Dis 80:32–34. doi:10.1016/j.diagmicrobio.2014.05.019 24952987

[17] Sissonen S, Pasanen T, Salmenlinna S, Vuopio-Varkila J, Tarkka E, Vaara M, Tissari P. 2009. Evaluation of a commercial MRSA assay when multiple MRSA strains are causing epidemics. Eur J Clin Microbiol Infect Dis 28:1271–1273. doi:10.1007/s10096-009-0771-z 19633873

[18] Belmekki M, Mammeri H, Hamdad F, Rousseau F, Canarelli B, Biendo M. 2013. Comparison of Xpert MRSA/SA nasal and MRSA/SA ELITe MGB assays for detection of the *mecA* gene with susceptibility testing methods for determination of methicillin resistance in *Staphylococcus aureus* isolates. J Clin Microbiol 51:3183–3191. doi:10.1128/JCM.00860-13 23863569 PMC3811630

[19] Laurent C, Bogaerts P, Schoevaerdts D, Denis O, Deplano A, Swine C, Struelens MJ, Glupczynski Y. 2010. Evaluation of the Xpert MRSA assay for rapid detection of methicillin-resistant *Staphylococcus aureus* from nares swabs of geriatric hospitalized patients and failure to detect a specific SCC*mec* type IV variant. Eur J Clin Microbiol Infect Dis 29:995–1002. doi:10.1007/s10096-010-0958-3 20512518

[20] Uehara Y. 2022. Current status of staphylococcal cassette chromosome *mec* (SCC*mec*). Antibiotics 11:86. doi:10.3390/antibiotics11010086 35052963 PMC8772726

[21] Sabat AJ, Bathoorn E, Chlebowicz-Fliss MA, Akkerboom V, Kamphuis I, Dos Santos CO, Friedrich AW. 2021. Misidentification of meticillin-resistant *Staphylococcus aureus* by the Cepheid Xpert MRSA NxG assay, the Netherlands, February to March 2021. Euro Surveill 26:37. doi:10.2807/1560-7917.ES.2021.26.37.2100800 PMC8447827 34533121

[22] Monecke S, König E, Earls MR, Leitner E, Müller E, Wagner GE, Poitz DM, Jatzwauk L, Vremerǎ T, Dorneanu OS, et al.. 2020. An epidemic CC1-MRSA-IV clone yields false-negative test results in molecular MRSA identification assays: a note of caution, Austria, Germany, Ireland, 2020. Euro Surveill 25:25. doi:10.2807/1560-7917.ES.2020.25.25.2000929 PMC7331142 32613938

[23] Mura T, Aso M, Kawamura K, Kanaya K, Ueda N, Iinuma Y. 2025. Case report: false-negative Xpert MRSA/SA BC result due to *spa* gene deletion in a patient with methicillin-resistant *Staphylococcus aureus* bacteremia. J Infect Chemother 31:102780. doi:10.1016/j.jiac.2025.102780 40769297

[24] Tenover FC, Tickler IA, Le VM, Dewell S, Mendes RE, Goering RV. 2019. Updating molecular diagnostics for detecting methicillin-susceptible and methicillin-resistant *Staphylococcus aureus* isolates in blood culture bottles. J Clin Microbiol 57:11. doi:10.1128/JCM.01195-19 PMC6813022 31484703

[25] Yarbrough ML, Warren DK, Allen K, Burkholder D, Daum R, Donskey C, Knaack D, LaMarca A, May L, Miller LG, Parenti DM, Peterson L, Widen R, Hernandez DR, Wolk DM, Burnham CA, Thean Yen Tan. 2018. Multicenter evaluation of the Xpert MRSA NxG assay for detection of methicillin-resistant *Staphylococcus aureus* in Nasal Swabs. J Clin Microbiol 56:e01381-17. doi:10.1128/JCM.01381-17 29118165 PMC5744196

[26] Boundy S, Zhao Q, Fairbanks C, Folgosa L, Climo M, Archer GL. 2012. Spontaneous staphylococcal cassette chromosome *mec* element excision in *Staphylococcus aureus* nasal carriers. J Clin Microbiol 50:469–471. doi:10.1128/JCM.01063-11 22116150 PMC3264175

[27] Enright MC, Robinson DA, Randle G, Feil EJ, Grundmann H, Spratt BG. 2002. The evolutionary history of methicillin-resistant *Staphylococcus aureus* (MRSA). Proc Natl Acad Sci USA 99:7687–7692. doi:10.1073/pnas.122108599 12032344 PMC124322

[28] Wielders CL, Vriens MR, Brisse S, de Graaf-Miltenburg LA, Troelstra A, Fleer A, Schmitz FJ, Verhoef J, Fluit AC. 2001. Evidence for in-vivo transfer of *mecA* DNA between strains of *Staphylococcus aureus*. Lancet 357:1674–1675. doi:10.1016/s0140-6736(00)04832-7 11425376

[29] Wang H, Hecht S, Kline D, Leber AL. 2019. *Staphylococcus aureus* and methicillin resistance detection directly from pediatric samples using PCR assays with differential cycle threshold values for corroboration of methicillin resistance. J Microbiol Methods 159:167–173. doi:10.1016/j.mimet.2019.01.009 30826439

